# Transcatheter PDA recanalization and interatrial septal stenting as a bridge to arterial switch operation in a late-presenting infant with D-transposition of the great arteries

**DOI:** 10.1186/s43044-025-00662-y

**Published:** 2025-06-26

**Authors:** Revan Satrio, Priyandini Wulandari, Hiradipta Ardining, Brian Mendel, Indriwanto Sakidjan Atmosudigdo, Radityo Prakoso, Bambang Widyantoro

**Affiliations:** 1 Department of Cardiology and Vascular Medicine, National Cardiovascular Centre of Harapan Kita, Universitas Indonesia, Jakarta, Indonesia; 2 Division of Pediatric Cardiology and Congenital Heart Disease, Department of Cardiology and Vascular Medicine, National Cardiovascular Centre of Harapan Kita, Universitas Indonesia, Jakarta, Indonesia; 3 Division of Acute Cardiovascular and Intensive Care, Department of Cardiology and Vascular Medicine, National Cardiovascular Centre of Harapan Kita, Universitas Indonesia, Jakarta, Indonesia

**Keywords:** Arterial switch operation (ASO), d-TGA/IVS, Late presentation, Left ventricular, Regression, Recanalization

## Abstract

**Background:**

Dextro-transposition of the great arteries with intact ventricular septum (d-TGA/IVS) requires early arterial switch operation (ASO) to preserve left ventricular function, but delayed presentations complicate outcomes due to LV regression and hypoxemia. Alternative bridging strategies are essential for late-presenting patients to improve surgical feasibility.

**Case report:**

We present a six-month-old male patient with dextrocardia, situs inversus, d-TGA/IVS who experienced persistent cyanosis despite prior balloon atrial septostomy (BAS). On admission, the patient exhibited severe hypoxemia (SpO₂ 33%), metabolic acidosis, and LV regression (LV mass index: 36–41 g/m2). Echocardiography confirmed a restrictive atrial septal defect (3.5 mm) and the absence of a patent ductus arteriosus (PDA). Given the prohibitive risk of immediate ASO, an emergency transcatheter intervention was performed. PDA recanalization was attempted. Following successful wire passage, balloon angioplasty and stent deployment restored systemic-to-pulmonary shunting, improving oxygen saturation to 56%. To further augment intercirculatory mixing, a 10.0 mm × 29 mm Omnilink Elite stent was implanted across the interatrial septum, increasing oxygen saturation to 85%. The patient demonstrated stable post-procedural hemodynamics and was subsequently bridged to elective ASO, which was performed successfully after two months.

**Conclusion:**

Transcatheter PDA recanalization and interatrial septal stenting represent a viable bridge to ASO in late-presenting d-TGA/IVS patients. This minimally invasive approach expands treatment options in resource-limited settings where early surgical intervention is not always feasible.

**Supplementary Information:**

The online version contains supplementary material available at 10.1186/s43044-025-00662-y.

## Introduction

Dextro-transposition of the great arteries with intact ventricular septum (d-TGA/IVS) is a life-threatening congenital heart defect, with an estimated prevalence of 4,000 live births per year [1]. The arterial switch operation (ASO) is the preferred corrective procedure, optimally performed within the first two weeks to one month of life [2]. After one month, the LV undergoes involution, making it unsuitable for ASO due to insufficient systemic pressure capacity [1, 2].

Despite this critical time frame, access to ASO is often limited, resulting in many patients presenting late in our country. Late presenters (aged > 1 month) are at risk of LV dysfunction, severe hypoxemia, and cyanosis, all of which compromise ASO outcomes [3, 4]. Early palliative surgical interventions such as modified Blalock–Taussig shunt (BTS) or pulmonary artery (PA) banding remain challenging in our setting, primarily due to limited infrastructure and the lack of a cardiac surgeon available to perform these procedures [5].

Transcatheter approaches have emerged as promising alternatives. Unlike cardiac surgery, which is concentrated in specialized centers, cath labs are more widely available, expanding access to non-surgical interventions [5, 6]. PDA stenting, combined with balloon pre-dilation, has been successfully performed as a bridging strategy. However, in d-TGA/IVS cases without a PDA, PDA recanalization offers a novel approach to reestablishing systemic-to-pulmonary circulation [6, 7]. We report a case of a late-presenting d-TGA/IVS patient who underwent emergent PDA recanalization and stenting, followed by IAS stenting, as a life-saving bridge to elective ASO.

## Case report

A 6-month-old, 5.9-kg male infant was admitted to our outpatient clinic with persistent cyanosis since birth, exacerbated by crying. At the previous hospital, he was diagnosed postnatally with D-TGA/IVS. Due to the scarcity of the cardiologist, Rashkind procedure was not performed and was delayed until the age of 2 months. He was only referred to our center at 6 months of age due to delays caused by the lengthy queue in the tiered referral system. Antenatal history revealed that the patient’s mother, a 34-year-old woman, had undergone routine antenatal visits. There was a history of one miscarriage. The infant was delivered at term via Cesarean section due to prolonged labor, with a birth weight of 3400 g. Although the baby cried immediately after birth, cyanosis was noted, leading to NICU admission.

**Fig. 1:**
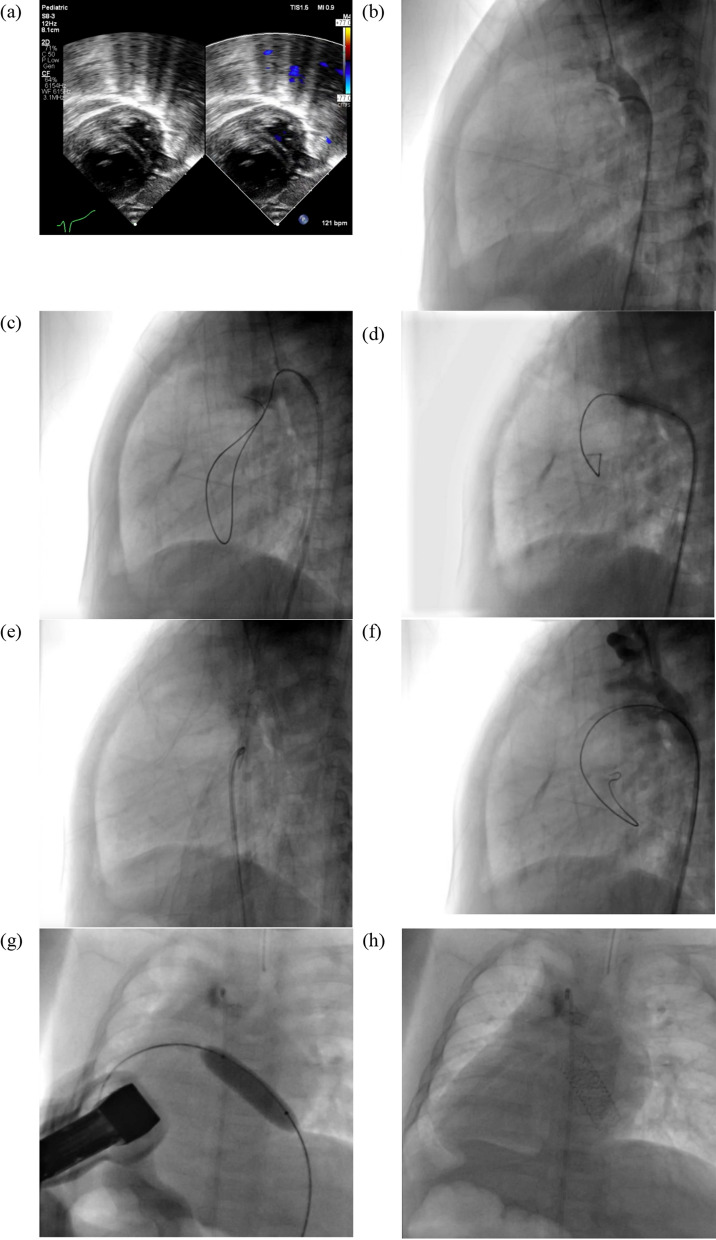
Ductal Recanalization and IAS Stenting in d-TGA/IVS. (**a**) Echocardiography showed an LVMI of 36–41 g/m2 and a D-shaped left ventricle, indicating higher right ventricular pressure. (**b**) Angiography revealed a completely occluded PDA with no pulmonary artery flow. (**c**) A V-18″ wire was successfully advanced into the right pulmonary artery. (**d**) A 3.0 × 15 mm Emerge balloon was inflated at 12 atm for 5 s. (**e**) A 4.0 × 16 mm Promus Premier stent was deployed at 16 atm, flared at 18 atm. (**f**) Post-procedure angiography confirmed proper stent placement with restored flow. (**g, h**) A 10.0 × 29 mm Omnilink Elite stent was deployed in the interatrial septum at 14 atm for 5 s

On admission, the heart rate was 151 bpm, respiratory rate of 35 breaths per minute, and peripheral oxygen saturation of 33%. Cardiac auscultation revealed a single accentuated second heart sound at the right upper parasternal area without murmurs. Echocardiographic findings included dextrocardia, situs inversus, D-TGA/IVS, post-BAS Atrial Septal Defect (ASD) measuring 3.5 mm, no Patent Ductus Arteriosus (PDA), Left Ventricular Mass Index (LVMI) of 36–41 g/m2, and a D-shaped left ventricle, suggesting right ventricular pressure exceeded left ventricular pressure (Fig. 1a). During observation, an episode of symptomatic bradycardia occurred, necessitating full resuscitation and mechanical ventilation. Blood gas analysis revealed metabolic acidosis with a lactate level of 5.4 mmol/L. Given the high surgical risk, emergency PDA recanalization with stenting was planned.

During procedure, right femoral arterial access was obtained using a 4/5F slender sheath, and an initial dose of intravenous (IV) heparin (350 IU) was administered intra-sheath. A 3.5/4F internal mammary artery (LIMA) catheter was advanced into the descending aorta (AoD), and selective angiography revealed a minute PDA stump with no contrast flow into the pulmonary artery (PA), indicating total occlusion from the mid-PDA (Fig. 1b). A stepwise recanalization approach was employed. Initial attempts with a 0.014″ Fielder XT wire failed to cross the occluded PDA. A 0.014″ ASAHI Gaia Second wire successfully crossed into the PA but recoiled back into the aortic arch (AoA) upon catheter advancement. Subsequently, a V-18″ wire was directed into the PDA and successfully advanced into the right pulmonary artery (RPA), yet catheter progression remained challenging (Fig. 1c). Reattempting with the ASAHI Gaia Second 0.014″ CTO wire enabled successful wire passage into the PA and left ventricle (LV), allowing for the exchange of the diagnostic catheter for a 3.5/5F JR guiding catheter.

Balloon angioplasty was performed using a 2.5 mm × 15 mm SAPPHIRE II balloon at 12 atm; however, the balloon ruptured due to overinflation and was removed. A subsequent 3.0 mm × 15 mm Emerge balloon was inflated at 12 atm for 5 s (Fig. 1d), followed by successful deployment of a 4.0 mm × 16 mm Promus Premier stent at 16 atm (Fig. 1e) with a flare technique at 18 atm. Post-procedure angiography confirmed adequate stent positioning with contrast flow into the PA (Fig. 1f). Oxygen saturation improved from 33 to 56%. However, this remained insufficient for a duct-dependent mixing lesion, where a minimum saturation of 75% is required.

Given the patient’s age and the limitations of BAS alone, interatrial communication was further augmented via IAS stenting. Right femoral venous access was obtained with a 6F sheath, subsequently upsized to 8F. A 3.5/5F JR guiding catheter was advanced through the inferior vena cava (IVC) into the right atrium (RA) and then into the left atrium (LA) using a 0.035″ Terumo wire. A 0.002 Amplatz 0.0035″ wire was positioned and anchored in the LA, allowing for catheter withdrawal. A 10.0 mm × 29 mm Omnilink Elite vascular stent was then deployed within the interatrial septum under fluoroscopic and echocardiographic guidance, inflated at 14 atm for 5 s. TTE and fluoroscopic evaluation confirmed optimal stent placement (Fig. 1g and h). Post-IAS stenting, peripheral oxygen saturation improved to 85%.

Post-procedure hemodynamic assessment revealed stable vitals. The patient recovered uneventfully and was transferred from the Cardiac ICU to the general ward on day four, maintaining a peripheral oxygen saturation of 85%. Post catheterization we give aspirin 30 mg OD as the only antiplatelet, captopril 6.25 t.i.d, spironolactone 6.25 mg OD, and furosemide 6 mg OD.

On follow-up, no clinical or echocardiography sign of pulmonary hypertension were found. Echocardiography showed left ventricular mass index (LVMi) of 61–65 g/m2 and LVPWd was 4.3 mm. Previously, his LVMi was 36–41 g/m2 and had a D-shaped left ventricle. Two months after catheterization, the patient underwent an arterial switch operation (ASO) with successful outcomes. During ASO, the interatrial septal stent was removed, and atrial septal defect was ultimately closed. At the most recent follow-up, the ASO demonstrated no postoperative complications or residual defects. Additionally, there were no clinical or echocardiographic signs of pulmonary hypertension in the patient. Postarterial switch operation, we give cefixime 30 mg b.i.d, furosemide 6 mg OD, captopril 3.125 mg t.i.d, and paracetamol 100 mg t.i.d.

## Discussion

### Left ventricular (LV) conditioning through recanalization of an atretic PDA

In patients with transposition of the great arteries (TGA) and an intact ventricular septum (IVS), the left ventricle (LV) is exposed to low pulmonary pressure, leading to regression and loss of systemic function. Arterial switch operation (ASO) is standard within the first month if the left ventricular mass index (LVMI) exceeds 40 g/m2 and the left ventricular posterior wall (LVPW) thickness is more than 3 mm [1, 2, 7]. However, in late presenters, such as our six-month-old patient, LV regression often precludes immediate ASO.

Traditional LV retraining involves pulmonary artery (PA) banding with a Blalock–Taussig (BT) shunt but carries risks like low cardiac output syndrome, prolonged intensive care days, and non-physiological afterload [6, 7]. Given these concerns, a transcatheter alternative was pursued. This approach aimed to (1) ensure sufficient preload for LV conditioning and (2) optimize intracardiac mixing for systemic oxygenation. Ductal stenting was planned to create an unrestricted patent ductus arteriosus (PDA), maintaining LV training and enabling a delayed ASO [6, 7, 9]. However, our case showed no ductus arteriosus, therefore recanalization was pursued.

Recanalization of a completely occluded ductus arteriosus presents significant technical challenges, particularly in the pediatric population. A stepwise wire escalation strategy was employed, starting with a 0.014" Fielder XT wire, which was unsuccessful in crossing the PDA. Ultimately, success was achieved using an ASAHI Gaia Second 0.014″ CTO wire, despite initial wire recoil. The use of high-support wires facilitated successful passage into the pulmonary artery, enabling subsequent balloon angioplasty and stenting. This highlights the applicability of advanced wire techniques commonly used in CTO interventions to complex congenital heart lesions interventions. Notably, our intervention established a functional aorto-pulmonary communication in the absence of a native PDA, resembling the physiological outcome of a Reverse Potts Shunt (RPS) [10].

PDA recanalization is rarely reported and challenging due to the absence of prograde flow (“probe patent” vs. “flow patent” ductus arteriosus). Kampmann et al. [11] first described PDA stenting in a 1.8 kg neonate with Tetralogy of Fallot, right aortic arch, and an isolated left pulmonary artery. Kothari et al. [7] later reported ductal recanalization and stenting in six patients aged 3–6 months with TGA/IVS, achieving adequate mixing and LV retraining in five cases. Failure occurred in one infant without a clear ampulla or ductal notch. Our findings suggest recanalization success may depend on ductal anatomy. Cardiac surgery following PDA stenting is generally safe, though stent removal may not always be possible, sometimes requiring additional pulmonary artery maneuvers. It is important to consider ductal recanalization in select cases, particularly when anatomical features suggest a higher probability of success. Ideally, the stent should fully cover the entire length of the ductus arteriosus. At the time of the procedure, the shortest available stent with an appropriate diameter was 16 mm, which was sufficient to span the entire duct. The development of microvascular plugs (MVP) has enabled transcatheter deliverable endoluminal pulmonary flow restrictor, potentially replacing PA banding. Haddad et al. (2023) showed that 28 PFRs were implanted in 14 patients where the patients experienced a significant drip in oxygen saturation and Qp/Qs. This approach enables stage-2 palliation or biventricular repair with lower risk by postponing surgeries to later infancy[8]. However, we did not have any experience with performing endovascular pulmonary banding. During follow-up, there were no signs of stenosis or other complications involving the pulmonary artery.

## The role of interatrial septal stenting

In congenital heart disease, an adequately sized and unrestricted atrial septal defect (ASD) is often critical for maintaining hemodynamic stability. In cases of dextro-transposition of the great arteries (d-TGA) with an intact ventricular septum (IVS), fetal echocardiography may identify a restrictive ASD, prompting consideration of balloon atrial septostomy (BAS) antenatally. BAS facilitates interatrial mixing, reduces left atrial pressure, and stabilizes neonates prior to an arterial switch operation (ASO), particularly when the ASD measures < 4 mm [12].

BAS is most effective in neonates younger than six weeks, as the interatrial septum remains thin and pliable, allowing for an effective tear. However, the procedure fails in up to 20% of cases due to incomplete balloon inflation or insufficient force during withdrawal, which results in septal stretching rather than definitive tearing. In patient with restrictive ASD, implantation of Atrial Flow Regulator could also improve interatrial mixing and serve as a bridge to definitive repair [13]. However, we did not consider AFR, as the device was unavailable at our institution and required a large sheath, which carries a higher risk of acute limb ischemia (ALI).

In our case, the patient was a late presenter at six months of age, by which time the atrial septum had thickened, presenting an additional procedural challenge. In such cases, the efficacy of BAS is further diminished, often necessitating PGE1 to counteract inadequate septostomy. However, ductal stenting and recanalization can serve as an alternative strategy, obviating the need for prolonged PGE1 administration [3, 12].

BAS carries potential complications, including balloon rupture, failure of balloon deflation, atrial perforation, and injury to adjacent structures, which can be promptly identified using echocardiography. Additionally, in older infants, the septum demonstrates an increased tendency to heal post-procedure, limiting the long-term efficacy of BAS. To ensure sustained and optimal interatrial mixing, we implanted a 10 mm Omnilink Elite stent, exceeding the expected 5 mm ASD size following septostomy. Stenting offers a more predictable and durable interatrial communication, particularly in patients with a thickened septum, and requires a smaller balloon diameter, minimizing the risk of transient heart block. However, potential complications include stent malposition, migration, fracture, thrombosis, and injury to adjacent structures [3, 12]. We utilized the formula whereby the IAS stent length was set to one-half to two-thirds of the combined length of the left and right atria (LA + RA) to ensure optimal stability. We have experiences with implanting shorter stents, which carry a higher risk of dislodgement. Although longer stents pose a risk of protrusion, they offer greater stability, and so far, we have not encountered any cases of stent protrusion.

Echocardiographic guidance is essential for precise stent placement, ensuring appropriate alignment within the atrial septum. The stent is delivered through a transseptal sheath positioned in the left atrium, gradually exposed, and adjusted prior to full deployment. A slight central constriction is maintained to create a “dog bone” or “butterfly” configuration, optimizing flow dynamics. Post-deployment imaging is performed to confirm stent stability, patency, and the absence of significant gradients or obstruction to pulmonary venous or inferior vena cava (IVC) flow. While longer stents may increase the risk of atrial erosion or thrombosis, careful patient selection and close echocardiographic follow-up are essential to mitigate these risks [3, 12].

## Conclusion

In conclusion, PDA recanalization and IAS stenting offer a viable transcatheter approach to bridge late-presenting d-TGA/IVS patients to arterial switch operation. This strategy restores systemic-to-pulmonary circulation, facilitates LV conditioning, and optimizes oxygenation, providing an alternative to conventional LV retraining.

## Supplementary Information

Additional file 1.

Additional file 2.

Additional file 3.

Additional file 4.

Additional file 5.

Additional file 6.

Additional file 7.

Additional file 8.

Additional file 9.

## Data Availability

No datasets were generated or analyzed during the current study.

## Abbreviations

ASD: Atrial septal defect
BAS: Balloon atrial septostomy
d-TGA: Dextro-transposition of the great arteries
IAS: Interatrial septum
IVC: Inferior vena cava
IVS: Intact ventricular septum
LV: Left ventricle
PDA: Patent ductus arteriosus

## References

[1] van der Palen RLF, Blom NA, Kuipers IM, Rammeloo LAJ, Jongbloed MRM, Konings TC et al (2021) Long-term outcome after the arterial switch operation: 43 years of experience. Eur J Cardiothorac Surg 59(5):968–977. 10.1093/ejcts/ezab006.PMID:33942860;PMCID:PMC8106945 33942860 10.1093/ejcts/ezab006 PMC8106945

[2] Ladouceur M, Ruperti-Repilado FJ, Rutz T (2023) Arterial switch operation: a surgical triumph with long-term management challenges. Int J Cardiol Congenit Heart Dis 8(15):100487. 10.1016/j.ijcchd.2023.100487.PMID:39713497;PMCID:PMC11657405 10.1016/j.ijcchd.2023.100487 PMC11657405 39713497

[3] Prakoso R, Kurniawati Y, Siagian SN, Sembiring AA, Sakti DDA, Mendel B et al (2024) Inter-atrial septum stenting in congenital heart disease patient: a case series in Indonesia. Cardiovasc Hematol Disord Drug Targets 24(3):163–171. 10.2174/011871529X320825240925073605.PMID:39411958;PMCID:PMC11826880 39411958 10.2174/011871529X320825240925073605 PMC11826880

[4] Pletzer S, Atz A, Chowdhury S (2019) The relationship between pre-operative left ventricular longitudinal strain and post-operative length of stay in patients undergoing arterial switch operation is age dependent. Pediatr Cardiol 40(2):366–373 30413855 10.1007/s00246-018-2018-1 PMC6415533

[5] Prakoso R, Simanjorang CNS, Kurniawati Y, Mendel B, Rahmat B, Zahara R et al (2024) Ductal stenting vs. surgical shunting in late presenting duct-dependent pulmonary circulation: a single-center experience. Front Cardiovasc Med. 11:1382879. 10.3389/fcvm.2024.1382879 38707893 10.3389/fcvm.2024.1382879 PMC11066280

[6] Ahmed A, LaCroix GA, Bader Ishqair AH, Shivaram P, Das S (2020) Late transcatheter recanalization of a closed ductus arteriosus in a 2-month-old infant with tetralogy of Fallot and isolated left pulmonary artery. Ann Pediatr Cardiol 13(4):357–360. 10.4103/apc.APC_168_19 33311929 10.4103/apc.APC_168_19 PMC7727898

[7] Kothari SS, Ramakrishnan S, Senguttuvan NB, Gupta SK, Bisoi AK (2011) Ductal recanalization and stenting for late presenters with TGA intact ventricular septum. Ann Pediatr Cardiol 4(2):135–138. 10.4103/0974-2069.84651.PMID:21976872;PMCID:PMC3180970 21976872 10.4103/0974-2069.84651 PMC3180970

[8] Haddad RN, Bentham J, Hassan AA, Soufi MA, Jaber O, Rassi IE et al (2023) Outcomes of manually modified microvascular plugs to pulmonary flow restrictors in various congenital heart lesions. Front Cardiovasc Med. 10.3389/fcvm.2023.1150579 37492157 10.3389/fcvm.2023.1150579 PMC10363685

[9] Leong MC, Alhassan AAA, Sivalingam S, Alwi M (2019) Ductal stenting to retrain the involuted left ventricle in D-transposition of the great arteries. Ann Thorac Surg 108(3):813–819. 10.1016/j.athoracsur.2019.03.045 30998905 10.1016/j.athoracsur.2019.03.045

[10] Mendel B, Christianto C, Angellia P, Holiyono I, Prakoso R, Siagian SN (2022) Reversed Potts shunt outcome in suprasystemic pulmonary arterial hypertension: a systematic review and meta-analysis. Curr Cardiol Rev 18(6):e090522204486. 10.2174/1573403X18666220509203335.PMID:35538823;PMCID:PMC9893152 35538823 10.2174/1573403X18666220509203335 PMC9893152

[11] Kampmann C, Wippermann CF, Schmid FX (1998) Transcatheter recanalisation and stenting of a closed ductus arteriosus in duct-dependent lung perfusion. Heart 80(2):206–207 9813575 10.1136/hrt.80.2.206 PMC1728785

[12] Mishra J (2022) Atrial septostomy and atrial septal stenting: role of echocardiography. J Indian Acad Echocardiogr Cardiovasc Imaging 6(3):181–185. 10.4103/jiae.jiae_9_22

[13] Bichali S, Delarue A, Houeijeh A (2024) Atrial flow regulator in transposition of the great arteries, ventricular septal defect, and pulmonary stenosis: a case report. Cardiol Young 34(10):2240–2243. 10.1017/S1047951124026258 39354847 10.1017/S1047951124026258

